# Supplementary material for: Iron and Nitrogen-Doped Wheat Straw Hierarchical Porous Carbon Materials for Supercapacitors
Source: Nanomaterials (Basel). 2024 Oct 23;14(21):1692. doi: 10.3390/nano14211692 (PMC11547934; doi:10.3390/nano14211692)
Supplement: Supplementary file 1 [file nanomaterials-14-01692-s001.zip › nanomaterials-3268640-supplementary.pdf]

Iron and Nitrogen-Doped Wheat Straw Hierarchical Porous Carbon Materials for Supercapacitors

Xiaoshuai Sun, Xiangyu Chen, Jiahua Ma, Chuanshan Zhao *, Jiehua Li and Hui Li

State Key Laboratory of Biobased Material and Green Papermaking, Qilu University of Technology,
Shandong Academy of Sciences, Jinan 250353, China;
17861407148@163.com(X.S.);colacxy@163.com(X.C.);13290394238@163.com (J.M.); lijiehua1995@163.com (J.L.); 15106900618@163.com (H.L.)
* Correspondence: ppzcs78@163.com(C. Zhao).

S1. Material Characterization:

Transmission electron (JEM-2100, 200 kV) and scanning electron (Zeiss EVO-18, 10 kV) microscopes (TEM and SEM, respectively) were used to observe microstructures. SSA and pore size distribution data were collected using a Kubo X1000 device and processed using Brunauer–Emmett–Teller (BET), Horvath–Kawazoe (HK), and Barrett–Joyner–Halenda (BJH) calculation methods. X-ray powder diffraction (XRD) measurements were carried out at room temperature using a Bruker D8 Advance X-ray powder diffractometer with Ni-filtered Cu-Ka radiation (λ=0.154 nm) from 5º-80º. The elemental composition of the samples was determined via X-ray photoelectron spectroscopy (XPS, Thermo Fisher Scientific). Raman spectra were obtained using a micro-Raman setup with a LabRAM Aramis microspectrograph at a wavelength of 532 nm (2.34 eV).

S2. Electrochemical performance characterization

The electrochemical performance in the three-electrode system was evaluated

using a CHI760E electrochemical workstation (Shanghai Chenhua Instrument Co., Ltd.). Cyclic voltammetry (CV) was performed over a potential range of -1 to 0 V, with scan rates of 5, 10, 20, 50, and 100 mV s^{-1}. Galvanostatic charge–discharge (GCD) measurements were conducted at current densities of 0.5, 1.0, 2.0, 5.0, and 10.0 A g^{-1}. The electrical impedance spectroscopy was carried out over a frequency range of 0.01 to 100 kHz with an amplitude of 5 mV.

Based on information gathered from the GCD test, the mass specific capacitance (C_m) of the sample was computed as follows:

$$C_m = \frac{I \times \Delta t}{m \times \Delta V} \tag{S1}$$

where m is the mass of electrode active material (g), δt is the discharge period (s), I is the constant discharge current (A), and δV is the potential difference after the IR drop is removed during the discharge time. The following formula can be used to assess the supercapacitor's charge storage mechanism:

$$i = kv^b \tag{S2}$$

$$log(i) = b\,log(v) + log(k) \tag{S3}$$

where the response current i (A g-1), measured at a fixed voltage, is exponentially related to the scanning rate v (m V-1) and has a power exponential relationship with the scan rate v (mV s-1) and k and b are constants. The formula for calculating the contribution value of the capacitance (surface control capacitance and diffusion control capacitance) is as follows:

$$i = k_1 v + k_2 v^{1/2} \tag{S4}$$

where $k_1 v$ and $k_2 v^{1/2}$ correspond to the capacitance behavior generated by the fast kinetic

process and the diffusion behavior generated by the slow kinetic process, respectively. The pseudocapacitance associated with the Faradaic charge transfer process governs the diffusion behavior, where k1and k2 are constants.

The electrochemical performance was evaluated using an electrochemical workstation, while the cycle stability was assessed with a LAND battery tester in the two-electrode system test. The potential window was set from 0 to 1 V, the current density range for GCD was 0.5 to 10.0 A g^{-1}, and the scan rate range for CV was 5 to 100 mV s^{-1}. The mass specific capacitance (C_s) of the symmetrical supercapacitor device was calculated based on data obtained from the GCD test as follows:

$$C_s = \frac{I \times \Delta t}{M \times \Delta V} \tag{S5}$$

where M is the mass (in grams) of the two electrodes' active material; all other symbols have the same meaning as previously described. The energy density E (Wh kg-1) and power density P (W kg-1) of the supercapacitor device were calculated using the following formulas during the two-electrode test:

$$E = \frac{1}{2} \times C_s \times \Delta V^2 \times \frac{1}{3.6} \tag{S6}$$

$$P = E/\triangle t \times 3600 \tag{S7}$$

S3 XPS profile of WSC-Fe/N-800

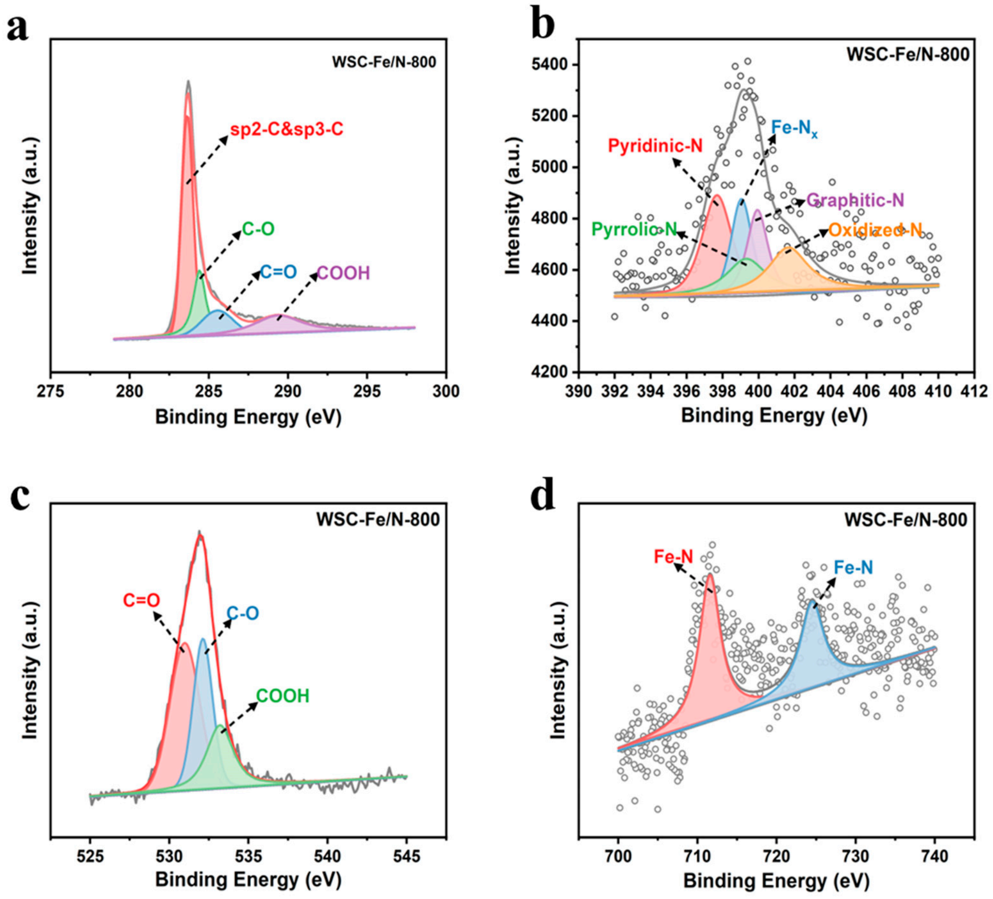

Figure S1 (a-c) C 1s, (c) O 1s, (d) N 1s, and (d) Fe 2p for WSC-Fe/N-800

S4 XPS profile of WSC-Fe/N-1000

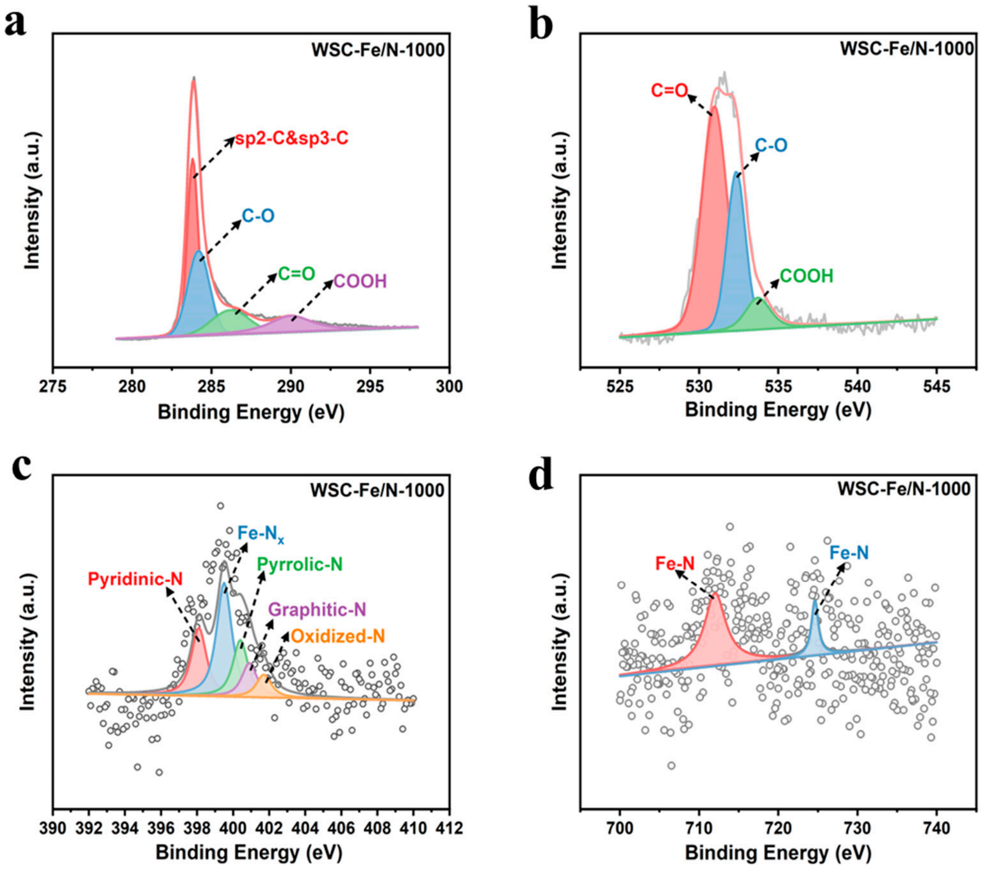

Figure S2 (a-c) C 1s, (c) O 1s, (d) N 1s, and (d) Fe 2p for WSC-Fe/N-1000

S5 Specific surface area of all samples

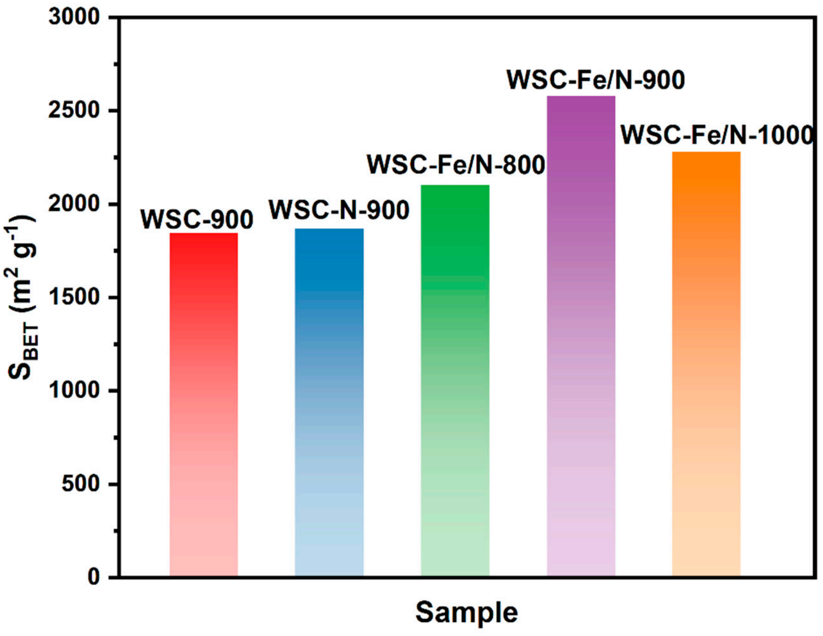

Figure S3 Specific surface area of all samples

S6. Materials

The wheat straw utilized in this study was sourced from a company in Shandong Province, China. Ferric chloride hexahydrate ($FeCl_3 \cdot 6H_2O$), potassium hydroxide (KOH, electronic grade, 99.99%), and ammonium chloride (NH_4Cl, analytical reagent grade, 99.5%) were purchased from Shanghai Aladdin Biochemical Technology Co., Ltd. All chemicals used in the study were of analytical grade and were applied without further purification.

Table S1. Surface composition and the content of different nitrogen doping configurations of samples.

Sample	C(at%)	O(at%)	Fe(at%)	N(at%)					
				Pyridinic-N	Fe-N	Pyrrolic-N	Graphiti-cN	Oxidize-dN	Total
**WSC-Fe/N-800**	**86.83**	**9.47**	**0.71**	**0.90**	**0.56**	**0.42**	**0.53**	**0.57**	**2.98**
**WSC-Fe/N-900**	**82.91**	**11.65**	**0.82**	**1.26**	**1.71**	**0.71**	**0.71**	**0.21**	**4.61**
**WSC-Fe/N-1000**	**86.47**	**10.57**	**0.46**	**0.57**	**0.96**	**0.48**	**0.29**	**0.19**	**2.50**

Table S2. Comparison of performance of carbon materials for SCs.

Sample	Electrolyte	C（$F\ g^{-1}$）	REF
**WSC-Fe/N-900**	**6 M KOH**	**400.5 $F\ g^{-1}$（0.5 $A\ g^{-1}$）**	**This work**
**AMH**	**2.5 MKNO3**	**275 $F\ g^{-1}$（0.5 $A\ g^{-1}$）**	**36**
**TGC-600**	**6 M KOH**	**199 $F\ g^{-1}$（0.2 $A\ g^{-1}$）**	**33**
**CC-220**	**6 M KOH**	**133.32 $F\ g^{-1}$（1 $A\ g^{-1}$）**	**37**

**PELAC**	**6 M KOH**	**336 F g^{-1}（1 A g^{-1}）**	**38**
**SRs-900**	**6 M KOH**	**350.2 F g^{-1}(0.2 A g^{-1})**	**35**
**micro-and mesoporous carbons**	**1 M H2SO4**	**195 F g^{-1}(0.1 A g^{-1})**	**39**
**starch-derived mesoporous carbons**	**6 M KOH**	**144 F g^{-1}(0.05 A g^{-1})**	**40**
**nitrogen-doped, ordered mesoporous carbons**	**6 M KOH**	**230 F g^{-1}(0.5 A g^{-1})**	**42**
**nitrogen-doped carbon**	**6 M KOH**	**252 F g^{-1}（0.5 A g^{-1}）**	**41**
**nitrogen-doped hierarchical porous carbon**	**6 M KOH**	**275 F g^{-1}（0.2 A g^{-1}）**	**16**
